# A Potential Role Exists for Nutritional Interventions in the Chronic Phase of Mild Traumatic Brain Injury, Concussion and Sports-Related Concussion: A Systematic Review

**DOI:** 10.3390/nu15173726

**Published:** 2023-08-25

**Authors:** Tansy Ryan, Sarah Nagle, Ed Daly, Alan J. Pearce, Lisa Ryan

**Affiliations:** 1 Department of Sport Exercise & Nutrition, Atlantic Technological University, Dublin Road, H91 T8NW Galway City, Galway, Ireland; tansy.ryan@atu.ie (T.R.); ed.daly@atu.ie (E.D.); 2 College of Sport, Health and Engineering, La Trobe University, Plenty Road and Kingsbury Drive, Melbourne, VIC 3086, Australia; alan.pearce@latrobe.edu.au

**Keywords:** concussion, nutrition, diet, supplement, recovery

## Abstract

Mild traumatic brain injury (mTBI) represents a significant burden for individuals, economies, and healthcare systems worldwide. Recovery protocols focus on medication and physiotherapy-based interventions. Animal studies have shown that antioxidants, branched-chain amino acids and omega-3 fatty acids may improve neurophysiological outcomes after TBI. However, there appears to be a paucity of nutritional interventions in humans with chronic (≥1 month) symptomology post-mTBI. This systematic literature review aimed to consolidate evidence for nutrition and dietary-related interventions in humans with chronic mTBI. The review was registered with the International Prospective Register of Systematic Reviews (PROSPERO; CRD42021277780) and conducted following the Preferred Reporting for Systematic Reviews and Meta-Analyses (PRISMA) guidelines. Three reviewers searched five databases (PubMed/MEDLINE, Web of Science, SPORTDiscus, CINAHL Complete and Cochrane), which yielded 6164 studies. Nine studies met the inclusion criteria. The main finding was the lack of interventions conducted to date, and a quality assessment of the included studies was found to be fair to good. Due to heterogeneity, a meta-analysis was not feasible. The six nutrition areas identified (omega-3 fatty acids, melatonin, Enzogenol^®^, MLC901, ketogenic diet and phytocannabinoids) were safe and well-tolerated. It was found that these nutritional interventions may improve cognitive failures, sleep disturbances, anxiety, physical disability, systolic blood pressure volume and sport concussion assessment tool scores following mTBI. Potential areas of improvement identified for future studies included blinding, reporting compliance, and controlling for confounders. In conclusion, further research of higher quality is needed to investigate the role of nutrition in recovery from mTBI to reduce the burden of chronic outcomes following mTBI.

## 1. Introduction

Traumatic brain injuries (TBIs) are often referred to as the ‘silent epidemic’. This is because many injuries go unrecognised and are excluded from epidemiological data [1]. These injuries represent a significant global health burden and substantial cost to economies and healthcare systems [2]. It has been estimated that the cost of new TBI incidence is USD 47.9 million in the first year. At the individual level, they can impact the long-term health of the person and cause disability. Epidemiological data estimate the incidence of TBI at 27.08 million cases annually, with age-standardised incidence rates at 369 per 100,000 population [3]. Other statistics suggest that incidence may be as high as 790 cases per 100,000 person-years [4]. However, it is suggested that the true incidence of TBIs may even be higher [5] as underreporting has been suggested at a factor of 6 to 10 times [6].

### 1.1. Traumatic Brain Injury—Definition and Diagnosing

Traumatic brain injuries can be categorised according to severity: mild, moderate, and severe injury, as defined by the Glasgow Coma Score (GCS) [7]. A mild traumatic brain injury (mTBI) is typically diagnosed with an initial Glasgow Coma Scale (GCS) of 13–15, loss of consciousness for up to 30 min, post-traumatic amnesia for less than 24 h and usually, the absence of positive neuroimaging or skull fractures [8]. The Centre for Disease Control and Prevention defines an mTBI as “a complex pathophysiological process affecting the brain, induced by traumatic biomechanical forces secondary to direct or indirect forces to the head” [9]. These physical forces can cause neurological dysfunction and neural cell death and may induce secondary long-lasting injuries [10]. In contrast, moderate and severe injuries are classified with a lower GCS and positive neuroimaging or skull fractures [4,11]. Although mTBIs account for 95% of TBIs and are 18 times more likely to occur, research focuses primarily on the severe category [4,11,12,13].

Often, the terms “mTBI” and “concussion” are used interchangeably, which is incorrect. Concussions are subsets of mTBI, and while all concussions are mTBIs, not all mTBIs are concussions [14]. Although the term concussion has been documented in sports for many years, it has become increasingly synonymous with sports recently. Noteworthy, only 15.5% are sports-related concussions (SRCs) [15]. Sport-related concussion refers to a TBI caused by biochemical forces to the head or surrounding areas experienced during a sporting activity [16]. A common feature of SRC is the rapid onset of neurological impairment, which tends to resolve spontaneously. Some of the acute symptoms are suggestive of a functional disturbance rather than a physical injury, which is reflected in negative neuroimaging [16,17]. The risk of SRC is highest in collision sports. Among the most common of these are American football, rugby, soccer and Australian Rules football [15]. Recent research found that the rate of concussion was 0.06 concussions per 1000 h. Additionally, players were 78 times more likely to sustain one during a match versus training [18].

### 1.2. Symptoms and Quality of Life Following mTBI

In those with mTBI, approximately three-quarters of people have symptom resolution within 4 weeks of injury. Almost all of these patients recover fully within 2 months [19,20]. However, recovery is not always straightforward. Some individuals can experience persistent symptoms or a collection of these symptoms, known as post-concussion syndrome (PCS) [21] or, more recently, as persistent post-concussion symptoms (PPCSs) [22]. Persistent symptoms can manifest as headaches, fatigue, dizziness, motion and light sensitivity, anxiety, depression, memory loss, personality changes and cognitive impairment [23]. Chronic symptoms are not the only aspect of individuals’ lives that may become affected following injury. Individuals’ quality of life (QoL) may be disrupted including relationship breakdown, unemployment, and disconnect from society [24]. Also, there is a growing concern that chronic and repeated mTBIs are associated with negative long-term outcomes [25,26].

### 1.3. Is There a Role for Nutrition in Recovery?

While it is well known that diet plays an essential role in maintaining neurological function, little research has focused on the role of nutrition in mTBI recovery and the alleviation of PCS symptoms [27,28]. Most research advises rest and medical and physiotherapy-led interventions in acute and chronic recovery [16,29]. Notably, nutrition was not included in the 2017 concussion consensus statement [16]. This systematic literature review (SLR) is based on the recent review by McGeown et al. [30], which detailed some promising results for antioxidants, creatine, omega-3 fatty acids (n-3FAs), multi-supplements, and the ketogenic diet in animal models following mTBI injury [31]. The aim of this review was to systematically consolidate evidence regarding the role of nutritional strategies in the chronic phase of mTBI in adult humans. Specifically, our question to be answered regarded adults with PCS/PPCS—do nutrition-related interventions, compared to placebo-control, relevant medication control or no comparator, improve recovery?

## 2. Materials and Methods

This systematic review was registered with the International Prospective Register of Systematic Reviews (PROSPERO) before commencing the search (registration number CRD42021277780). The review was conducted following the Preferred Reporting for Systematic Review and Meta-Analyses (PRISMA) guidelines (see Appendix A) [32]. As outlined in Table 1, the review methodology was developed as per the population, intervention, comparator and outcome (PICO) model.

### 2.1. Study Search and Selection Criteria

The five electronic databases that were chosen for this review were PubMed/MEDLINE, Web of Science, SPORTDiscus, CINAHL Complete and Cochrane Reviews. The review was limited to studies published from January 1998 to 2023 to emulate the work of McGeown et al. [30]. Also, it was reasoned that research on mTBI is constantly evolving, and most of the relevant research was published within the last 25 years. Both researchers (SN and LR) agreed on search terms for the review (Table 1). Group 1 and 2 terms were based on the terms used in McGeown et al. [30], with the addition of recovery terms (Group 3) selected from medical subject headings.

### 2.2. Study Selection

Eligible studies for this review were (1) peer-reviewed Randomised Controlled Trials (RCTs) and observational studies; (2) studies on adults (≥18 years), (3) studies in the chronic phase of mTBI, concussion or SRC, where chronic was defined as symptoms lasting at least 1 month, (4) studies that included a measure of recovery, (5) studies that included a nutrition-related or dietary-related intervention(s) administered in the chronic phase of injury and (6) studies in English. The exclusion criteria included (1) animal or cell studies, (2) studies exclusively in children and/or adolescents (aged ≤ 17 years), (3) studies exclusively on moderate and/or severe TBI, (4) studies on acute concussion (symptoms lasting less than 1 month), (5) non-nutrition or dietary-related intervention(s), (6) dietary-interventions with parenteral and/or enteral nutrition and (7) student theses, case reports, conference abstracts, limited review book chapters. The study designs that were eligible for inclusion were observational, descriptive and qualitative interventions, such as randomised trials, non-randomised trials, case-control studies, non-case-control studies, cohort studies and pilot studies. If applicable, SLRs were used to hand-search reference lists. Narrative reviews were excluded.

### 2.3. Data Extraction

The initial search and extraction were conducted in 2020. The search protocol was re-run and updated in 2023. EndNote V20 was used to collate the search results into a universal database. Two researchers (SN and LR) agreed on the exclusion criteria. Studies were excluded and coded accordingly if they were not a population, intervention, outcome, study or language of interest. Any queries or disagreements regarding the selected studies were discussed and, if required, a third reviewer contributed to the final decision. After screening based on titles and abstracts, full-text studies were then reviewed and assessed for inclusion. All studies that met the inclusion criteria were hand-searched to identify studies that may have been missed in the original search. The extracted data were then defined by the following headings: author, country of study, study design, participant characteristics, participant baseline and intervention data, intervention details, measures assessed and outcomes (see Table 2). The level of evidence was assessed based on the National Health and Medical Research Council guidelines [33].

### 2.4. Quality Assessment

The quality of the included studies was assessed using a modified Black and Downs quality rating tool [43]. This tool has a checklist of 27 questions with “yes”, “no”, “partially” and “unable to determine” answers. The questions are organised into 5 topics: study quality (questions 1–10), external validity (questions 11–13), internal validity and bias (questions 14–20), internal validity, confounding and selection bias (questions 21–26) and power of the study (question 27). In this modified version of the quality tool, question 27 was adapted so that the studies were rated on whether they had sufficient power to detect clinically important effects. If a study had sufficient power, it was awarded 1 point and if otherwise, no points were awarded. Accordingly, the maximum number of points to be awarded was 28 instead of 32. Table 3 details the classification list as seen in [44]. This modified version has been used in previous SLRs [44,45,46,47].

## 3. Synthesis of Results

Due to the variety of nutritional therapies, participant characteristics and recovery outcomes across the studies, it was not possible to combine the quantitative data to conduct a meta-analysis. Thus, the findings are presented in narrative form. Table 2 details the study and participant characteristics. Table 4 outlines the main findings, and Table 5 summarises these findings for use by practitioners.

### 3.1. Article Selection and Quality Assessment

**Figure 1:**
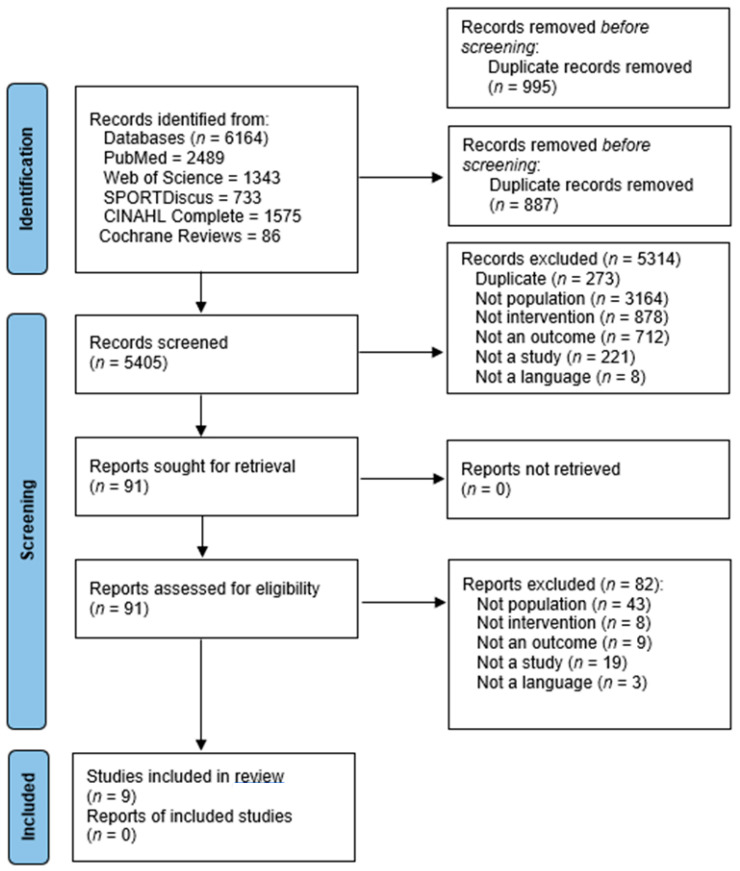
PRISMA flow diagram showing the included studies [32].

Figure 1 illustrates the PRISMA flow diagram. The initial search identified 6164 articles using database searches. Following the title and abstract screening, 91 entire papers were retrieved and assessed for inclusion, with 9 being eligible for inclusion [34,35,36,37,38,40,48]. Walton et al. [41] was excluded in the final review stage because it was unclear whether the participants (ex-National Football League players with a history of SRC) had chronic symptomology. Another paper [49] was excluded because it was unclear if participants had chronic symptoms. The participants had to make contact within 10 days of initial injury, and the intervention lasted 3 months. The majority of included studies were conducted after 2011 [34,35,37,38,40,41,48]. Of the nine studies, five were randomised-controlled trials (RCTs) [36,37,38,48]. Five papers had level II evidence [36,37,38,39,41,48,49] and three papers had level III evidence [34,35,40].

### 3.2. Details of the Included Studies and Participants

The causes of mTBI in the studies analysed included road traffic accidents, SRCs, workplace accidents, falls and assaults. Two studies consisted entirely of a sporting population [34,39]. Theadom et al. [38] included six participants, from a total of 60, that had a sports-related injury. The study by Amen et al. [34] did not specify the severity of TBI. Both researchers from our group agreed to classify these as SRCs because the participants were retired NFL players who had experienced brain damage and/or cognitive impairment from numerous TBIs sustained while playing football. Moreover, most studies included participants with a history of mTBI or concussion exclusively. Some included moderate and severe TBIs in the overall cohort studied [36,37,48]. One study included post-menopausal women who were medically diagnosed by their physician as having PCS. Though the timeframe between experiencing a concussion and when the investigation took place was not directly stated in the paper, upon contacting the authors, it became clear that this study was still eligible for inclusion in this SLR [42]. Regarding the length of time since injury, the inclusion criteria ranged from at least one month to three years post-injury.

### 3.3. Participant Characteristics

Participants in RCTs ranged from 7 to 78, with an average of 47. In this review, commonly reported chronic symptoms were cognitive difficulties, functional disabilities, mood disorders and sleep disturbances. Two of the studies included male-only participants [34,36]. Grima et al. [37] included 67% male participants. Three studies included a relatively even gender split: Theadom et al. [38] had 43% male participants, Walter et al. [39] had 48%, and Theadom et al. [48] had 50%. The remaining studies by Fotuhi et al. [35] and Rippee et al. [40] had a majority of female subjects, with 35% male and 14% male participants, respectively. One study recruited all female subjects [42]. As the inclusion criteria were specific to adults only, all studies that included paediatric-only populations, classified as those less than 18 years old, were excluded. Two studies had a mixture of paediatric and adult populations [35,36].

### 3.4. Length of Follow-Up

The studies consisted of a variety of follow-up intervals. In two studies, the follow-up was 12 weeks [35,40]. Two studies, which investigated the role of melatonin, had a 10-week follow-up interval [36,37]. Another study had 4-month interval [38]. The remaining studies had 6-week [39], 6 month [48], 2–12 month [34] and 10–28 day [42] follow-up intervals. 

As per Table 4, there were a variety of outcomes measured such as cognition, memory, attention, mood, motivation, sleep quality, QoL, balance and sport concussion assessment tool (SCAT) scores. Cognition and everyday memory were measured using the Microcog Assessment of Cognitive Functioning [34], Cognitive Failures Questionnaire [38,48], Central Nervous System Vital Signs [35,48] and Impact of Event Scale—Revised tool [40]. Other tests used to assess working memory were the Wechsler Adult Intelligence Scale and California Verbal Learning Test [38]. QoL was assessed using an adapted version of the Medical Outcomes Study 36-Item Short-Form Health Survey (SF-36) tool in Grima et al. [37]. In Theadom et al. [48], the “QoL after Brain Injury” tool was used to assess self-autonomy, cognition and physical domains of QoL. Mood disorders were measured using a range of questionnaires. The most frequently used tool to assess anxiety and depression was the Hospital Anxiety and Depression [36,37,38,48]. Other tools used were the General Anxiety Disorder and Patient Health Questionnaire and the SCAT tool [40,42].

Post-concussion symptoms were measured using the Rivermead Post-Concussion Symptoms Questionnaire [38,48] and the Post-Concussion Symptom Scale [40] Balance was assessed using a modified Balance Error Scoring System [40]. To assess sleep quality, the Epworth Sleepiness Scale was used in two studies to measure daytime sleepiness [35,37]. Sleep diaries that monitored sleep onset latency, duration, sleep quality and daytime alertness were kept in Kemp et al. [36]. The results were computed into averages for analysis. The Pittsburgh Sleep Quality Index was used to assess sleep onset latency and quality [37]. The Fatigue Severit Scale, and a modified version of this tool, was used by Grima et al. [37] and Theadom et al. [48], respectively, as an objective measure of fatigue during daily activities. Walter et al. [39] used electroencephalogram and virtual reality devices to assess brain activity during testing and spatial memory, reaction time and attention. One study included in-house assessments that were non-validated and may limit the generalisability of their findings [35]. Fotuhi et al. [35] used two in-house questionnaires that assessed lifestyle factors associated with brain health, neurocognitive and neurobehavioural symptoms associated with PCS/PPCS.

### 3.5. Details of Interventions and Findings

The most common intervention was dietary supplementation with a nutrient or commercial product. The included nutritional interventions can be divided into six areas: studies involving n-3FAs, melatonin, Enzogenol^®^, MLC901, dietary manipulation in terms of the ketogenic diet (KD) and CBD or a CBD:THC formula.

#### 3.5.1. Studies Involving Omega-3 Fatty Acids

Two studies used n-3FA supplements in different dosages for chronic [34,35]. Those in Amen et al. [34] were given a fish oil supplement that contained 1720 mg eicosapentaenoic acid (EPA) and 1160 mg docosahexaenoic acid (DHA), a multiple vitamin and a “brain enhancement supplement” daily. The doses of the two latter products were not reported and compliance data were not available. Also, the participants received education regarding brain health advice such as smoking cessation, exercise and substance abuse. There was an improvement in cognition and brain perfusion, self-reported mood, motivation, sleep and memory after intervention. However, it was not possible to ascertain whether these findings were attributable to the n-3FAs, the other supplements or the synergistic relationship between all three products and the education element. As part of the multifaceted CRP analysed by Fotuhi et al. [35], the participants consumed n-3FA supplements containing 1000–1500 mg of EPA and DHA daily and followed a Mediterranean-style diet. Similarly, compliance data were not reported. Additionally, the participants had access to a “brain coach” for advice regarding exercise, stress relief and education. Notable results were improvements in cognition, attention, memory, executive functioning and reaction time.

#### 3.5.2. Melatonin

Melatonin was included as a nutrition-related intervention because it is often referred to as a dietary supplement and is sold over the counter and in health food shops internationally. It was used in two studies to investigate its potential to improve disturbances post-TBI [36,37]. Notably, both studies included a spectrum of TBI severities and had two participants with mTBI. Each used a randomised, double-blind, controlled and cross-over design and chose the same follow-up period (10 weeks). Different doses of melatonin were used: 5 mg in Kemp et al. [36] versus 2 mg in Grima et al. [37]. The studies reported a reduction in anxiety; however, this was not significant in Kemp et al. [36]. Noteworthy, there were seven participants in total in this study, and no power calculation was provided. Also, melatonin was compared to amitriptyline, a known antidepressant, which may have reduced the study’s ability to produce significant findings. Grima et al. [37] found that melatonin was associated with a significant reduction in self-reported anxiety, *p* = 0.006. No significant difference was found in either study for depression. Grima et al. [37] reported a moderate and significant reduction in PSQI, which signified improved sleep quality. Interestingly, no significant association between TBI severity and treatment effect for sleep onset latency or PSQI were found. The use of actigraphy technology reported a significant increase in sleep efficiency. In contrast, no improvement in sleep quality, duration, latency or daytime alertness was reported after melatonin use [36].

#### 3.5.3. Enzogenol^®^

Enzogenol^®^ is a supplement associated with antioxidant and anti-inflammatory properties. Two RCTs used 1000 mg daily [38,39]. Theadom et al. [38] observed that self-reported cognitive shortcomings improved after supplementation, which was indicative of better daily cognition. However, it did not improve episodic memory. Its treatment effects stabilised after 11 weeks, and improvements in anxiety and depression levels were found after the intervention. This was the only study in this review to conduct a sensitivity analysis, and the results did not significantly change after a sensitivity analysis was conducted. Walter et al. [39] reported that Enzogenol^®^ improved self-reported sleep disturbances and mental fatigue. The supplement was generally well tolerated, except for some reports of blurred vision, sleep disturbances and headaches.

#### 3.5.4. The Ketogenic Diet (KD)

One study investigated the role of the KD in treating symptoms of chronic concussion [40]. The participants were instructed to consume a diet of 70–75% energy from fat, 20–25% energy from protein and 5–10% energy from carbohydrates. This study found that visual memory and symptoms of PCS/PPCS may benefit from 2 months of consuming this high-fat, low-carbohydrate diet. As this was a feasibility study, it was cautioned that these findings may have been coincidental and limited by the low number of participants (*n* = 11) who completed the trial. No significant changes were reported for depression, anxiety or postural stability. The KD required continuous monitoring and input from the researchers and a dietitian, which may limit feasibility and increase cost in a real-life setting.

#### 3.5.5. MLC901 Capsules

MLC901 capsules, which contain herbal components, such as radix astragali, prunus persica and radix polygalae, were used in Theadom et al. [48]. These are commonly used in Chinese medicine and have antioxidant properties. Adherence to the supplement remained over 85% at 6 months. Those who were randomised to the MLC901 group (*n* = 36) reported significant improvements in executive functioning and complex attention. These improvements slowed after 6–9 months of ceasing the treatment, which supports an ongoing treatment effect. No other significant differences were found for cognitive functioning, neurobehavioural sequelae of fatigue, mood or QoL. It is unclear if earlier administration of this product would have caused greater treatment effects, as most participants were 5–6 months post-injury. Overall, MLC901 was well-tolerated and safe for those with mild and moderate TBI. Some side-effects reported were headache, sore tongue and itchiness.

#### 3.5.6. Phytocannabinoids

Singh et al. [42] investigated the effects of phytocannabinoids on heart rate variability (HRV) and either low-frequency (LF) or high-frequency (HF) domain blood pressure variability (BPV) in post-menopausal females diagnosed with PCS by their physician. With participants self-administering either full spectrum CBD or a 20:1 CBD:THC formula under the guidance of their physician, the dosing varied widely. From the first to forth follow-up visits, participant 1 took 100 mg, 200 mg, 400 mg and 400 mg CBD, respectively; participant 2 took 100 mg CBD at visit 2 and 200 mg CBD at visits 3–5; participant 3 took 25 mg and 50 mg at visits 2 and 3–4, respectively; and finally, participant 4 took 40:2, 40:2, 20:1 and 24:1.2 mg CBD:THC at visits 2–5, respectively. For all participants, systolic HF BPV increased throughout the duration of the study. HRV did change, but not in the same pattern as seen for BPV. Though the LF/HF ratio decreased throughout each visit for participant 1, the opposite was observed for participants 2, 3 and 4. The authors suggested that this may be related to the increase in HF power for participant 1, while participants 2 and 4 experienced reduced HR power, and participant 3 experienced decreased LF power in comparison to their baseline measurements. Regarding SCAT scores, participants 1, 3 and 4 began with scores of 88, 47 and 8, respectively, while participant 2, who experienced a minor head injury 10 days prior to follow-up visit 3, was unable to quantify her symptoms using a Likert scale. Participant 1′s SCAT score dropped on visit 2 and began to increase from then onwards. Participant 3 and 4 both experienced a drop in SCAT scores from baseline to their final visit from 47 and 8 to 2 and 2, respectively. The authors consider their investigation on the effects of phytocannabinoids on HRV and BPV as the first of its kind to observe these beneficial effects. They concluded that administering phytocannabinoids had a beneficial effect on systolic BPV. However, with all participants administering various amounts, a personalised approach may be required for treatment rather than a blanket recommendation.

### 3.6. Quality and Risk of Bias Assessment

**Figure 2:**
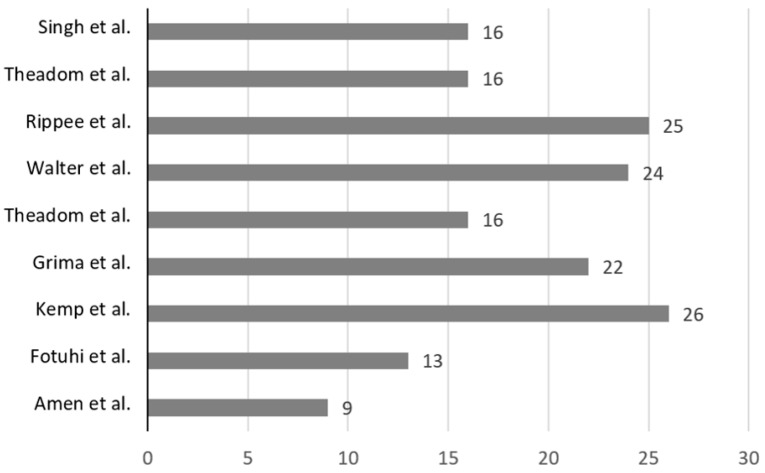
Visual representation of relative study quality, with study first author and year of publication, with the average value for the 9 included studies (18.6) [34,35,36,37,38,39,40,42,48].

The results of the modified Black and Downs [43] quality tool are summarised in Table 6 and Figure 2. The average score of the studies was 19. Four of the included studies had above average scores [36,37,38,48]. One study was deemed as “excellent” [37]. Two of the studies that investigated n-3FAs scored ≤14 [34,35]. Most studies were classified as “good” with scores between 20 and 25 [36,38,48]. Notably, questions 11 and 12, which assessed external validity, found that most studies did “not meet” the criteria or were “unable to determine” if the criteria was met. Questions 14 to 26 assessed the risk of bias (RoB). Most studies suitably blinded the participants and researchers (questions 14 and 15). Notably, all studies met the criteria that examined whether the main outcomes measured were accurate and reliable (question 20). Question 25 assessed if an adequate adjustment was made for confounding. It was reported that four studies did not meet this criterion, and one study was unable to be determined. Only one study in this review reported conducting a sensitivity analysis [48].

## 4. Discussion

This review highlighted the dearth of high-quality prospective interventions and clinical trials in the area of nutritional interventions for chronic mTBI in humans. The lack of evidence is surprising considering the promising results using animal models of mTBI [30,31]. In this review, a meta-analysis was not possible because of heterogeneity in treatments, which may have affected the strength of the generated findings.

### 4.1. Participant and Study Characteristics

It is generally accepted that a gender bias exists in the scientific literature, and this bias extends into the domains of sports science and neuroscience. Epidemiological data suggests that males account for most of the recorded TBI statistics [4,50]. In this review, both genders were represented, despite two studies with 100% male participants and one study with 100% female participants [34,36,42]. Emerging research suggests females tend to suffer worse symptoms and longer recovery time [51]. In this systematic review, studies did not examine if gender differences existed, with some suggesting that low participant numbers did not allow for this [39]. In terms of intervention administration, participants in most studies were fed ad libitum with the exception of Rippee et al. [40]. Although, this is representative of the habitual intake of the target audience of this review, it may have led to high variability in what was consumed outside of the interventions and possible nutrient interactions between other foods eaten. Notably, studies with artificial nutrition were excluded from the search, and although these would have allowed for less diversity in intake, the findings would have been more representative of those with moderate or severe injury.

### 4.2. The Possible Role of Nutrition in Recovery from mTBI

Despite limitations in the number of studies to date, the data provide novel findings regarding nutritional recovery for humans with chronic symptoms post-mTBI. To date, any knowledge relating to nutritional interventions has mainly been based on animal models. The nutritional areas examined in this review were associated with improved cognitive and memory function [34,35,38,40,48], better overall sleep quality [37,39], improved mood disorders [31,38] and a beneficial effect on systolic BPV and SCAT scores [42]. The common themes in these interventions were that they were feasible, well-tolerated and safe to use. Findings from preclinical trials have reported that n-3FAs in clinically high doses from 10 mg/kg/day to 370 mg/kg/day improved cognitive and neurological performance post-mTBI (pooled ES of 1.52–3.55) [31]. A case study in severe head trauma reported that 19,212 mg of n-3FAs daily with enteral feeding showed no adverse side effects [52]. Although these dosages are in excess of most national guidelines, they have been suggested in ‘real-life’ scenarios, such as in the National Collegiate Athletic Association, where 9000 mg/day of n-3FAs is recommended [31]. Due to differences in the studies investigating n-3FAs in this review, an optimal dosage in humans cannot yet be recommended.

Moreover, interest in antioxidant-based interventions has grown due to promising research suggesting that their administration following mTBI may ameliorate oxidative stress and inflammation and alter the clinical progression of these injuries [53]. This systematic review builds on the findings from a similar review in which antioxidants following TBI were associated with improved recovery and cognitive function, as well as reduced TBI sequelae and decreased mortality risk [54]. In addition, the finding that melatonin is associated with improved post-traumatic sleep quality is positive, as it is estimated that 50% of individuals following TBI experience sleep disturbance [55]. Its use may extend to the treatment of other PCS/PCSS symptoms, such as headaches [56]. In that cohort study, melatonin significantly improved post-concussion headaches in 75% of those taking it. The specific variables of interest included improved alertness, improved sleep duration, improved sleep quality and reduced latency. In addition, two studies [34,35] included in this review may provide evidence to suggest the benefits of combining nutritional interventions with either another nutrition-related supplement or a lifestyle modification. Previous studies have reported added benefits of multiple interventions in treating TBI [57,58]. The latter suggests a beneficial additive effect of combining supplements. In this case, the addition of curcumin to DHA significantly improved learning latency in rats following TBI [58]. The inclusion of concurrent dietary and exercise interventions was previously found to further compound the benefits of nutritional interventions [58,59].

In their 2023 review of nutrition for cognitive health, Puri et al. [60] highlight the evidence supporting various nutritional strategies that have the potential to protect against cognitive decline. Among these, micronutrients like iron and B groups, as well as high-protein and low-fat diets have shown a positive effect on maintaining cognitive health. Regarding the impact of dietary habits on sleep quality, many of the studies in this area are of poor-to-fair quality, making it a challenge to establish a definitive causal relationship. However, current research indicated that diets higher in processed and free-sugar rich foods are associated with a decrease in sleep quality [61]. Termed “nutritional psychiatry”, the concept of mood disorder treatment with nutritional practices is an emerging area of research. Though research is limited, Martins et al. [62], highlight that the findings of observational studies suggest that a high-quality diet, characterized as high in fruits, vegetables, legumes, nuts, whole grains and high-quality protein sources, have a counteractive effect on mood disorders, particularly major depressive disorder and bipolar disorder. A recent review reported more specific findings, noting that although more clinical trials are needed, probiotic supplementation seemed to be a positive add-on therapy for major depressive disorder treatment [63]. Research into specific nutrients and their mechanisms of action in this area is still a relatively new area with much work remaining until definitive recommendations can be established. Future research should focus on single nutrients, particularly protein, probiotics and those that are available in significantly higher levels in whole-food compared with highly processed diets.

The Black and Downs [43] quality tool is designed mainly for RCTs and non-RCTs, which may have led to these designs scoring higher. Most studies were considered fair- to good-quality, with these being primarily RCTs that detailed patient characteristics, study design and outcome measures, as well as reporting exact *p*-values. Some studies included a flow diagram to depict participant flow and attrition throughout the study, which enabled methodological transparency [37,38,48]. The RoB components in the quality tool identified areas that should be considered in future studies, such as blinding, selecting appropriate statistical tests, reporting compliance, adverse events, external validity and power. Notably, all studies reported a positive effect of the interventions on recovery outcomes, which may indicate possible publication bias.

### 4.3. Limitations

There were limitations in this review that are worth noting. All the research included in this review recruited adults, and thus, it should not be assumed that these findings may be generalized to children or adolescents. Similarly, the participants were in the chronic phase of mTBI, and additional research is required to investigate whether the same findings would apply during the acute phase of such injury. Heterogeneity may have impacted the strength of evidence. These differences included population variability, different dosage of nutrients, diversity of injury and outcome measurements. In addition to this, the variety of nutritional therapies in a small number of studies may have reduced the potential to find the most effective treatment. Thirdly, the search strategy had limitations because it excluded paediatric and adolescent populations, those with moderate and severe TBI, those in the acute phase of mTBI and those receiving enteral or parenteral nutrition. Thus, the results should not be generalisable to these populations. Moreover, limitations of the individual studies should be considered before interpreting the findings. Only a single study included in this review reported conducting a sensitivity analysis. The studies reported small sample size [35,36,39] and smaller than intended sample size [37], the absence of a control group [35,40] and the non-randomised, open-label design used by Amen et al. [34] as limitations to findings. Selection bias was included as a possible limitation in Theadom et al. [38].

### 4.4. Practical Applications and Future Directions

It is the role of a multidisciplinary team, in the clinical and high-performance setting, to ensure recovery is optimised for patients and athletes after mTBI or concussion. Table 5 may be useful for practitioners in this field. Due to inherent limitations within the evidence presented, it was not possible to suggest one nutritional intervention as the single most promising remedy for chronic mTBI. The evidence suggests the need for more research on humans to improve the biofidelity of these findings and to translate findings from animal models into real-life settings. These trials should ensure sufficient participant numbers and standardised protocols to provide clinically effective results. These trials should also consider determining the therapeutic dose of such interventions. Also, the possible role of nutrition as a prophylactic treatment for mTBI recovery is an interesting concept with some positive outcomes, such as improving learning disability as noted in animals [64]. Observational studies may provide interesting data in the context of pre-treating these injuries.

## 5. Conclusions

A main finding of this review is the lack of available evidence that examines the role of nutrition in chronic recovery from mTBI. This review contributes to previous animal research on the potential for nutritional interventions in treating chronic symptomology following brain injury. Dietary adaptations have the potential to play a role in improving cognitive failures, sleep disturbances and other mood disorders following injury as well as individual LF, BPV and SCAT scores. It is promising that these interventions are considered safe and tolerated in most humans. In conclusion, this review presents opportunities for future clinical trials to optimise recovery for those with PCS/PPCS, particularly in the provision of accessible “over-the-counter” solutions in an effort to reduce the global health burden of this condition.

## Figures and Tables

**Table 1:** PICO model and search strategy in accordance with PRISMA 2020 guidelines, where * indicates any potential ending to the phrase will also be searched [32].

Population (P)	Adults (≥18 years and older) that have had an mTBI, concussion or SRC, single or repetitive injury and have chronic symptoms (lasting ≥1 month)	
Intervention (I)	Nutrition and/or dietary-related interventions administered in the post-injury chronic phase	
Comparator (C)	Placebo-controlled, relevant medication control or nothing	
Outcomes (O)	Measure of recovery following intervention such as recovery from symptoms, return to work or return to sport	
Group 1: Concussion terms	Group 2: Nutrition or dietary-related intervention	Group 3: Recovery terms
postconcussion OR “post-concussion” OR concussion OR tbi OR mtbi OR “traumatic brain injur*” OR “cortical impact” OR “fluid percussion” OR “acceleration injury”	diet* OR supplement* OR “neuroprotective agent*” OR creatine OR antioxidant* OR “anti-oxidant*” OR “fatty acid*” OR vitamin* OR nutri* OR nutraceutical OR keto* OR “amino acid*”OR “complementary and integrative medicine” OR “complementary and alternative medicine”	recovery OR “symptom resolution” OR “symptom-free” OR “symptom free” OR restoration OR “return to play” OR “return to activity” OR improvement OR rehab* OR “treatment recovery” OR “recovery of function”

**Table 2:** Characteristics of the Included Studies.

Author (Year) Country	Study Design	Aims and Objectives	Type of Injury	Participant Characteristics				Follow-Up	Length of Chronic Symptoms	Intervention	Daily Dosage
				Baseline *n*, % Male	Background, Age	Follow-Up *n*, % Male	Sporting Population				
Studies involving Omega-3 Fatty Acids											
Amen et al. [34] USA	Non-randomised, open-label, single-arm study	To reverse brain damage and cognitive dysfunction in former NFL players.	SRC	*n* = 30, 100%	Retired NFL players who had recurrent traumatic head injuries and who had brain damage and cognitive impairment Age—NR.	NR	Yes	2–12 months	Retired players who had head injuries during their playing career	Multifaceted intervention with a dietary component and education on “brain healthy lifestyle”, such as stopping smoking, drug abuse, nutrition and exercise. Dietary components: Fish oil supplement; High potency multivitamin; Brain-enhancement supplement that contained ginkgo, vinpocetine, phosphatidylserine, ALC, huperzine, ALA and NAC.	EPA 1720 mg and DHA 1160 mg; NR; NR reported as “clinically effective doses”.
Fotuhi et al. [35] USA	Secondary analysis of a non-randomised, open-label, single-arm study	To perform a retrospective analysis of data collated from those who completed the NeuroGrow Programme (CRP) and to evaluate changes in neurocognition post intervention.	mTBI	*n* = 46, 35%	Those in the NeuroGrow CRP who had mTBI and experience PCS. Age (years): ≥10 Overall: 31.7 (15.9) *	NR	No	12 weeks	At least 3 months following the patient’s relevant injury	Multifaceted rehabilitation program with a dietary component and education from a “brain coach” regarding sleep, stress management, diet and exercise. Dietary components: Omega-3 fatty acid supplement; Advice regarding a Mediterranean diet (fruit, vegetables, wholegrains, olive oil and fish).	Omega-3 fatty acid containing 1000–1500 mg/day of DHA + EPA
Melatonin											
Kemp et al. [36] UK	Randomised, double-blind, medication- controlled, crossover trial	To explore the potential of melatonin as an alternative to medications in post-TBI sleep disturbance.	Mild to severe TBI	*n* = 7, 100%	Community-dwelling adults with sleep disturbance following TBI. mTBI; *n* = 2 (29%) Age (years): 17.2–54.7 Overall: 39.6 (NR) *	*n* = 7, 100%	No	10 weeks	At least 6 months post-injury. Group (months): 36.3 (NR) *	Intervention: Melatonin Control: Amitriptyline Methodology: Participants received the intervention or control treatment for 1 month, which was followed by a 2-week washout period. After this, participants swapped and received the other treatment for 1 month.	Intervention: Melatonin 5 mg Control: Amitriptyline 25 mg
Grima et al. [37] Australia	Randomised, double-blind placebo-controlled, crossover, clinical trial	To evaluate the effect of melatonin on those with sleep disturbance post-TBI.	Mild to severe TBI	*n* = 35 randomised, 67%	Community-dwelling adults with sleep complaint following TBI. mTBI; *n* = 2 (6%), in placebo group Age (years): 18–65 years Overall: 37 (11) * Intervention to control group: 35 (11) * Control to intervention group: 38 (10) *	*n* = 33, NR	No	10 weeks	Months post-injury: Overall: 46 (13–102) ** Intervention to control group: 61 (28–115) ** Control to intervention group: 25 (10–72) **	Intervention: Melatonin in two-piece gelatine capsule Control: Identical placebo capsule containing mannitol, acacia and pure icing sugar Methodology: Participants had a 2-week baseline run-in period, which was followed by two treatment periods lasting 4 weeks of either melatonin or the control. The initial treatment was followed by a crossover period of the alternative treatment. There was a 48 h washout period between treatments.	Intervention: Melatonin 2 mg Control: Mannitol (106 mg), acacia (11 mg) and pure icing sugar (106 mg)
Enzogenol^®^											
Theadom et al. [38] NZ	Pilot, randomised, double-blind, placebo-controlled, clinical trial	To explore the safety and feasibility of the recruitment and administration of Enzogenol^®^, to examine efficacy of it on memory and PCS symptoms and to examine the influence of FFM and diet on outcome changes following supplementation.	mTBI	*n* = 60 randomised, 43%	Community-dwelling adults with persistent cognitive difficulties following mTBI. Age (years): 21–64 Intervention group: 45 (27) ** Control Group: 44 (28) **	*n* = 50, NR	Partial, *n* = 6 (10%) sports-related injury	16 weeks	3–12 months post injury Months post injury: Intervention group: 71 (2.66%) * Control group: 8.04 (2.46%) *	Intervention: Enzogenol^®^ capsule Control: Microcrystalline cellulose placebo in an identical capsule Methodology: Participants were allocated to 1. Enzogenol^®^ (*n* = 30) or 2. placebo (*n* = 30) for 6 weeks. Then, both groups received Enzogenol^®^ for 6 weeks, which was followed by 4 weeks of the placebo.	Intervention: Enzogenol^®^ 1000 mg (2 × 500 mg capsules in the morning) Control: Microcrystalline cellulose 1000 mg (2 × 500 mg capsules in the morning)
Walter et al. [39] USA	Randomised, placebo-controlled, preclinical trial	To examine the effect of Enzogenol^®^ on chronic concussion.	SRC	*n* = 42, 48%	College-aged athletes with history of SRCs (≤5 SRCs) and residual chronic symptoms. Age (years): 18–24	*n* = 42, 48%	Yes	6 weeks	Those 6 months to 3 years post-injury	Intervention: Enzogenol^®^ capsule Control: Microcrystalline cellulose placebo in a dyed brown capsule identical to Enzogenol^®^ Methodology: Subjects were instructed to take either the intervention or control for the study duration.	Intervention: Enzogenol^®^ 1000 mg (2 × 500 mg capsules in the morning) Control: Microcrystalline cellulose 1000 mg (2 × 500 mg capsules in the morning)
The Ketogenic Diet (KD)											
Rippee et al. [40] USA	Pilot, non-randomised, open-label single-arm, feasibility study	To examine the feasibility of implementing KD in those with PCS symptoms and to investigate changes in self-reported symptoms and cognitive status.	Concussion	*n* = 14, 14%	Community-dwelling adults with PCS symptoms. Age (years): 18–65 Overall: 45 (12.2) *	*n* = 11, NR *n* = 12 completed the study. *n* = 1 was non-compliant and did not reach ketosis. They were excluded from analyses.	No	2 months	At least 4 weeks post-injury Days post-injury: 103 (56.0–217.5) **	Nutrition counselling from a registered dietitian to consume and monitor adherence to the KD.	Participants had to consume: KD at a 1:1 ratio of g lipid to g of non-lipid, energy as 5–10% from CHO, 70–75% from fat and 20–25% from protein and MCT oil ~1–2 tbsps.
Studies involving MLC901											
Theadom et al. [41] NZ	Pilot, randomised, double-blind, placebo-controlled, clinical trial	To test the efficacy and safety of MLC901 on cognition and to examine its effect on mood, fatigue, physical disability, QoL and neurobehavioural sequalae.	Mild and moderate TBI	*n* = 78, 50%	Adults post-mild and moderate TBI with cognitive difficulties. Mild and mod TBI: (*n* = 2, 3% mod) Age (years): 18–65 Overall: 37.5 (14.8) * Intervention: 38.6 (14.1) * Control: 38.4 (15.7) *	*n* = 50, NR	No	6 months	1–12 months post-injury	Intervention: The MLC901 capsule contained 9 herbs known for having antioxidant and anti-inflammatory properties Control: Made from dextrin and magnesium stearate, visually indistinguishable from the intervention pills Methodology: Subjects were instructed to take either the intervention (MLC901) or placebo for the duration of the study. Participants were assessed at 9 months.	Intervention: Two × 0.4 g capsules (0.8 g), three times Control: Two × 0.4 g capsules (0.8 g), three times
Studies involving CBD											
Singh et al. [42]	Double-blind randomised controlled trial	To investigate the responses of phytocannabinoid administration on blood pressure and heartrate variability in PCS women.	PCS	*n* = 4 0%	Females medically diagnosed with PCS. Age (years): 42–57 Overall: 31.7 (15.9) *	*N* = 4 0%	No	Every 10–28 days over a total of ≤70 days	Years	Multifaceted intervention with dietary component of self-administered CBD or a CBD:THC formula. Dietary components: Self-administered CBD or a 20:1 CBD:THC formula under physician guidance.	CBD 25–400 mg or 20:1 CBD:THC formula

* mean (standard deviation) ** median (interquartile range). Abbreviations (alphabetical order): CBD = cannabidiol; EPA = eicosapentaenoic acid; KD = ketogenic diet; NZ = New Zealand; PCS = post-concussion syndrome; QoL = quality of life; THC = tetrahydrocannabinol; UK = United Kingdom; USA = United States of America.

**Table 3:** Modified Black and Downs Quality Classification [43].

Level of Quality	Poor	Fair	Good	Excellent
Sum Score	≤14	15–19	20–25	26–28

**Table 4:** Summary of Outcomes Measured and Results.

Author (Year) Country	Level of Evidence ^a^	Dietary Intervention Specifics	Measurements	Results	Brief Summary of Results
Studies involving Omega-3 Fatty Acids (n-3fas)					
Amen et al. [34] USA	III	Omega-3 fatty acid, high-potency multiple vitamin and brain enhancement supplement	Micro-cognitive test known as the MACF. This test contained nine subtests that examined general cognitive functioning, cognitive proficiency, attention memory, reaction time, information processing speed and accuracy, spatial processing, reasoning and speed. Brain SPECT image analysis, a standard brain imaging tool, was used. It evaluated the effects of the interventions on brain function.	MACF reported improvements in post-intervention outcomes in seven of the nine subtests: - General cognition functioning 31.8 (24.1) * to 43.4 (25.7) *, *p* < 0.000; - General cognitive proficiency 24.7 (20.1) * to 35.2 (23.5) *, *p* < 0.000; - Processing speed 33.1 (24.8) * to 39.3 (25.5) *, *p* = 0.026; - Processing accuracy 40.9 (28.7) * to 48.5 (29.1) *, *p* = 0.012; - Attention 38.4 (26.2) * to 48.7 (27.6) *, *p* = 0.025; - reasoning 32.7 (25.7) * to 41.6 (28.0) *, *p* = 0.006; - Memory 33.8 (27.4) * to 42.9 (28.4) *, *p* = 0.022. Non-significant improvements were reported in spatial processing and reaction times. Brain SPECT scans showed significant increases in brain perfusion, where *p* < 0.001 (particularly in the prefrontal cortex, anterior cingulate gyrus, parietal lobes, occipital lobes and cerebellum). Participants reported increases in memory (69%), attention (53%), mood (38%), motivation (38%) and sleep (25%).	Dietary interventions with n-3fas, multivitamin and brain enhancement supplement, in conjunction with lifestyle modifications, may improve cognitive function and cerebral blood flow in those with a history of SRC.
Fotuhi et al. [35] USA	III	Omega-3 fatty acid supplement and dietary advice to follow a Mediterranean diet	The CNS vs. the assessed cognition, such as NCI, complex attention, reaction time, executive functioning, cognitive flexibility; composite, visual, working and verbal memory, sustained and simple attention; motor, processing and psychomotor speed. An in-house questionnaire (“brain fitness calculator”) examined factors associated with brain health, such as exercise, diet quality, Omega-3 supplementation compliance, social engagement, sleep quality, attitude and mood. Another in-house questionnaire (“concussion symptom questionnaire”) explored neurocognitive and neurobehavioural symptoms. A decrease in score suggested an improvement in symptoms. The ESS assessed daytime sleepiness in patients. A decrease in score indicated an improvement.	The NCI score, a summary of the CNS vs. tests, significantly improved from 85.3 (20.0) * to 95.8 (18.8) * post-treatment, *p* = 0.0001. This represented an improvement from a “low average” to an “average” score. A paired *t*-test reported a significant increase in NCI score of 10.6 (16.5) * points, *p* = 0.0001. This was a medium ES (0.64). Overall, 89% of subjects improved their NCI score post-treatment. Participants experienced significant rises in: (medium to large ES) - Complex attention 86.1 (27.8) * to 103.2 (11.8) *, *p* < 0.0001; - Executive functioning 85.9 (25.3) * to 103.9 (17.6) *, *p* < 0.0001; - Cognitive flexibility 85.1 (25.4) * to 103.2 (18.1) *, *p* < 0.0001; - Verbal memory 86.7 (18.8) * to 97.1 (20.3) *, *p* = 0.0004; - Psychomotor speed 91.5 (17.5) * to 98.2 (17.5) *, *p* = 0.0005; - Reaction time 80.9 (30.1) * to 91.1 (24.7) *, *p* = 0.0004; - Working memory 97.9 (19.6) * to 107.5 (11.0) *, *p* = 0.0013; - Sustained attention 99.3 (14.5) * to 106.0 (13.4) *, *p* = 0.0017. Non-significant improvements were reported for composite memory, visual memory, processing speed, simple attention and motor speed. ESS decreased from 8.6 (4.2) * to 6.9 (3.6) *. The mean change in score was 1.7, which was not significant after Bonferroni’s correction (*p* = 0.018). As measured using the reliable change method, 27% of patients had improvements for complex attention, 30% for cognitive flexibility and 33% for executive functioning. Results from the “brain fitness calculator” increased from 46.1 (8.7) * to 54.4 (9.4) * post-treatment (*n* = 38). Findings from the “concussion symptom questionnaire” decreased from 156.7 (65) * to 111.9 (58.9) * (*n* = 35).	Dietary interventions with n-3FAs, in conjunction with other lifestyle modifications, may improve markers of neurocognition and PCS symptoms in those with chronic concussion symptomology.
Melatonin					
Kemp et al. [36] UK	II	Melatonin or amitriptyline	Participants were to keep a sleep diary to assess sleep onset latency, duration, sleep quality and daytime alertness throughout the intervention. Results from these diaries were computed into averages. ES were calculated for each drug for all four sleep variables, i.e., improved alertness, improved sleep duration, improved sleep quality and reduced latency. Neuropsychological and mood data were assessed using the SCOLP, AMIPB (to examine information processing) and the HADS (self-reported anxiety and depressive symptomology).	There was no significant improvement in any sleep variable after melatonin or amitriptyline. A repeated measures ANOVA reported that no main effect was found (F(2, 48) = 0.98, *p* > 0.05). Also, the sleep variable × treatment interaction result was non-significant (F(2, 48) = 2.2, *p* > 0.05). The largest ES was found for improved sleep duration and amitriptyline (ES = 0.56). ES = 0.42 was found for improved daytime alertness and melatonin. There was a non-significant reduction in both groups for anxiety (*p* = 0.66) and depression (*p* = 0.97) following melatonin and amitriptyline. There was no significant change reported in neuropsychological or mood data as measured using the SCOLP or AMIPB. Two participants self-reported improvements in sleep duration and sleep quality. Three participants had improved ratings for sleep duration, latency and quality for both drugs compared to the baseline.	Melatonin may alleviate some sleep disorders and disturbances following TBI.
Grima et al. [37] Australia	II	Melatonin administered as a pill; the placebo was matched for appearance	The PSQI was used to assess sleep quality and onset latency (primary outcome). The other measures of sleep quality assessed were sleep efficiency, daytime sleepiness, the ESS and the FSS (assessed self-reported fatigue during daily activities). The HADS was used to assess self-reported anxiety and depressive symptomology. The SF-36 v1 was used to assess 8 facets of self-reported QoL.	Melatonin was associated with a significant and moderate (ES = 0.46) reduction in PSQI scores, where melatonin was 7.86 versus the placebo was 9.47, with a difference of −1.79 (95% CI −2.70 to −0.88, *p* ≤ 0.0001). This result indicated improved sleep quality when taking melatonin. No significant reduction in sleep onset latency was reported for melatonin (*p* = 0.23), and the treatment sequence did not significantly affect the primary or secondary outcomes. A correlational analysis did not report any significant association between TBI severity and treatment effect for PSQI or sleep onset latency (*p* > 0.05). Melatonin was associated with a small but significant increase in actigraphic sleep efficiency, ES = 0.28, *p* = 0.04. Melatonin was associated with significant reduction in self-reported anxiety of (95% CI) −1.15 (−1.97 to −0.34), *p* = 0.006. No difference was found for depression between groups, *p* = 0.68. Results from SF-36 found that melatonin was associated with significant improvements in mental health (TEE; 2.51, 95% CI: 0.58–4.42, ES = 0.23, *p* = 0.01) and self-reported vitality (TEE; 3.67, 95% CI: 0.36–6.98, ES = 0.35, *p* = 0.03).	Melatonin over a short period of time may improve sleep quality and anxiety following TBI
Enzogenol^®^					
Theadom et al. [38] NZ	II	Capsules of Enzogenol^®^ or placebo	Validated tests were used to examine cognitive functioning (mainly memory). The CFQ was used to measure everyday memory. The WAIS Version 3, Arithmetic WAIS Version 4 and Letter Number Sequencing subtests of the WAIS Version 4 were used to assess working memory. The CVLT assessed episodic memory. Post-concussion symptoms such as headaches, dizziness, nausea, restlessness, noise and light sensitivity, sleep disturbance, blurred vision and balance difficulties were measured using the RPQ. Mood disorders were examined using the HADS.	There was a significant reduction in self-reported cognitive shortcomings as measured using CFQ scores in the intervention group. Mean difference in CFQ for Enzogenol^®^ versus the placebo was −6.9 points (95% CI; −10.8 to −4.1). Those in the intervention group had a 28% change versus a 22% change in the placebo group. *p* values were not documented. Enzogenol^®^ was associated with self-reported cognitive failures until the results stabilised at week 11. The observed benefits were stable during the 4-week period following cessation of the treatment. There were no significant treatment effects on working or episodic memory at 6 weeks based on WAIS and CVLT scores. Mean levels of anxiety and depression reduced by a similar amount in both groups at 6 weeks. Among those with moderate and severe levels of anxiety at baseline, 31% in the placebo group and 54% in the intervention group improved to normal levels at 6 weeks. Among those with moderate and severe depression at baseline, 71% in the placebo group and 83% in the intervention group improved to normal levels at 6 weeks There was a trend towards improved frequency of post-concussion symptoms as measured using the RPQ. Although, *p*-values were not listed, these improvements were referred to as “not statistically significant”.	Enzogenol^®^ is a well-tolerated and safe supplement for use in humans, and it may improve the frequency of self-reported cognitive failures. An RCT was recommended to examine and determine its efficacy in more detail.
Walter et al. [39] USA	II	Two capsules of Enzogenol^®^ daily	A VR system was used to assess balance, spatial memory, reaction time and attention. A neuropsychological battery was used to assess neurological function, and it included tests such as the HVLT-R, BVMT-R, SDMT, Vigil/W Continuous Performance test, modified Digit Span Test, CTMT, PSU Cancellation Test and the SCWT (“Stroop test”). The SCWT was conducted three times: at the beginning (Stroop test 1), middle (Stroop test 2) and end (Stroop test 3) of the battery. The test was used to cause cognitive exertion and provide comparison data for the EEG (same task at different times). The Beatty fatigue rating scale was used at the beginning and end to examine changes in self-reported fatigue. An EEG assessed electrical brain activity in the frontal midline, parietal and occipital areas of the brain during the tests.	There was a significant increase in FMT in the Enzogenol^®^ group, which may suggest that treated subjects were able to allocate brain resources better and focus attention on demands during prolonged neuropsychological testing. Paired *t*-tests found significant increases in raw FFT power at 6 weeks for sitting eyes open (t(20) = −2.42, *p* < 0.05) in the Enzogenol^®^ group. Results of the Stroop test 1 (t(20) = −2.78, *p* < 0.01) and Stroop Test 3 (t(20) = 2.28, *p* < 0.05) also found significant results for the treatment group. There were non-significant changes in ΔPSD in the occipital region in both groups. The ΔPSD in the placebo group decreased from 77.6 (22.1) * to 74.9 (13.0) * following the study. In contrast, the ΔPSD in the treatment group increased from 76.9 (17.1) * to 78.2 (12.2). * There was a decrease in absolute EEG theta power for the parietal region in the Enzogenol^®^ group at 6 weeks. The placebo group had a significant increase in power on the Stroop Test 2: (t(19) = −2.01, *p* < 0.05). An increase in FMT and a decrease in theta power can indicate reduced mental fatigue. Overall, there was an increase in parietal theta power across groups. However, this increase was less prominent in the Enzogenol^®^ group. After the intervention, 65% of those in the Enzogenol^®^ group self-reported less mental fatigue and fewer sleep problems.	In individuals with a history of SRC and residual symptoms, Enzogenol^®^ may reduce mental fatigue and improve self-reported sleep issues.
The Ketogenic Diet					
Rippee et al. [40] USA	III	Nutrition counselling and KD in a ratio of 1:1 g of lipid to grams of non-lipid	The ImPACT was used at baseline and month 2 to assess cognitive performance. The PCSS was used to evaluate symptoms of concussion at baseline, month 1 and month 2. The PHQ-9 and GAD-7 were used to assess depressive and anxiety symptomology. Balance was assessed at baseline and month 2 using the M-BESS.	The visual memory domain of the ImPACT values reported significant improvements following the KD intervention. Scores increased from 56.0 (51.5–66.0) ** at baseline to 70.0 (64.5–79.5) ** post-intervention, where *p* = 0.02. This was a mean improvement of 12.2 points. Though not-significant, the PCSS scores reported a decrease from 33.0 (19.5–65.5) ** to 28.0 (13.5–50.0) ** from baseline to month 2, where *p* = 0.34, which may suggest improved symptomology. No significant changes were found in the PHQ-9, GAD-7 or M-BESS following the intervention.	The KD may be a feasible dietary treatment for PCS and help to improve some memory domains.
MLC901					
Theadom et al. [41] NZ	II	Two capsules of the MLC901 three times daily	The CNS vs. measured cognitive function (primary function) across eight cognitive domains (listed in Fotuhi et al. (2020)). Secondary outcomes were measured using the CFQ, RPQ and HADS. Another secondary outcome measure was the Modified Fatigue Impact Scale, which assessed levels of perceived physical, cognitive and psychosocial aspects of fatigue. Also, the QoL after Brain Injury measure was included to assess perceived satisfaction with cognitive, self, autonomy and physical domains of QoL.	MLC901 had significant improvements on complex attention and executive functioning. Complex attention improved by 11.88 (*p* = 0.04, *d* = 0.6), and executive functioning improved by 7.16 (*p* = 0.04, *d* = 0.4). ES were small to moderate No significant differences were reported for any of the other domains of cognitive functioning after 6 months. Significant linear regression models for cognitive outcomes reported that consistent, cumulative improvements were observed. The domain of complex attention for MLC901 was 7.64 ± 1.81 (SE), *p* = 0.0000 at 1 month, 7.58 ± 2.09, *p* = 0.000 at 3 months and 8.58 ± 2.47 (SE), *p* = 0.0005 at 6 months. There was a decline after stopping the treatment. The regression model reported cumulative improvements in the MLC901 group for executive functioning. While improvements were also seen in the placebo group, they were accelerated in the MLC901 group: 9.73 ± 1.66 (SE), *p* = 0.0000 at 1 month, 8.90 ± 1.79, *p* = 0.0000 at 3 months & 12.74 ± 1.99, *p* = 0.0000 at 6 months. Both groups experienced improvements across all secondary outcomes. No significant differences were found between the groups for neurobehavioural sequelae of mood, QoL or fatigue across the different time-points. Those in the MLC901 group had a significant improvement in the cognitive domain of QoL: mean difference of 1.62 and small ES (*d* = 0.1).	MLC901 appears to be safe and well-tolerated. It may help to improve domains of executive functioning and complex attention in individuals following mTBI or moderate TBI.
Phytocannabinoids					
Singh et al. [42]	III	CBD/20:1 CBD:THC formula supplement	Finapres NOVA was used to measure blood pressure. Finapres NOVA and a three-lead electrocardiograph were used to assess HRV. SCAT scores from baseline.	All participants had increases in systolic HF BPV: - Participant 1; 19.2 mmHg2 to 33.4 mmHg2 at 200 mg but dropped to 14.6 mmHg2 by visit 5 (400 mg). - Participant 2; 20.0 mmHg2 to 31.5 mmHg2 at visit 5 (200 mg). - Participant 3; 2.5 mmHg2 to 11.6 mmHg2 at visit 4 (50 mg). - Participant 4: from 7.6 mmHg2 to 38.0 mmHg2 at visit 3 (40:2 mg CBD: 2 mg THC).. HRV values did not change in the same pattern as BPV: - Participant 1: decreased LF/HF ratio at each visit; - Participant 2: increased LF/HF ratio from baseline; - Participant 3: increased LF/HF ratio from baseline; - Participant 4: increased LF/HF ratio from baseline.	Dietary interventions with CBD or a 20:1 CBD:THC formula supplement may improve systolic BPV but at varying doses for different individuals.

^a^ derived from the National Health and Medical Research Council (NHMRC) * mean (standard deviation) ** median (interquartile range). Abbreviations (al-phabetical order): ΔPSD = alpha-power spectral density; AMIPB = adult memory and processing battery; BPV = blood pressure volume; BVMT-R = Brief Visuospatial Memory Test—Revised; CBD = cannabidiol; CFQ = Cognitive Failures Questionnaire; CI = confidence interval; CNS = Central Nervous System Vital Signs; CTMT = Comprehensive Trail-Making Test; CVLT = California Verbal Learning Test; EEG = electroencephalography; ESS = Epworth Sleepiness Scale; ES = effect size; FFT = Fast Fourier Transform; FMT = frontal midline theta; FSS = Fatigue Severit Scale; GAD-7 = general anxiety disorder; HADS = Hospital Anxiety and Depression Scale; HF = high frequency; HRV = heart rate variability; HVLT-R = Hopkins Verbal Learning Test—Revised; ImPACT = Immediate Post-Concussion Assessment and Cognitive Testing; KD = ketogenic diet; M-BESS = Modified Balance Error Scoring System; MACF = Microcog Assessment of Cognitive Functioning; NCI = neurocognition index; NZ = New Zealand; PCS = post-concussion syndrome; PCSS = Post-Concussion Symptom Scale: PHQ-9 = Patient Health Questionnaire; PSD = Power Spectral Density; PSQI = Pittsburgh sleep quality index; QoL = quality of life; RPQ = Rivermead Post-Concussion Symptoms Questionnaire; SCAT = sport concussion assessment tool; SCOLP = Speed and Capacity of Language-Processing Test; SCWT = stroop colour word tests; SDMT = the Symbol–Digit Modalities Test; SE = standard error; SF-36 = Medical Outcomes Study 36-Item Short-Form Health Survey; TEE = treatment effect estimate; THC = tetrahydrocannabinol; UK = United Kingdom; USA = United States of America; WAIS = Wechsler Adult Intelligence Scale; VR = virtual reality.

**Table 5:** Summary of main outcomes.

Study	Summary
Amen et al. [34]	Participants: Retired athletes with cognitive impairment and who experienced recurrent TBIs or SRCs during their playing career. Intervention: n-3FAs (1720 mg EPA and 1160 mg DHA), multivitamin and brain-enhancement supplement (containing antioxidants) with education for up to 1 year. Outcomes: Improvement in neurocognitive outcomes, increased brain perfusion and self-reported increases in mood, memory, motivation, sleep and attention.
Fotuhi et al. [35]	Participants: Community-dwelling individuals with PCS. Intervention: Multidisciplinary CRP with a dietary component (omega-3 FA containing 1000–1500 mg/day of DHA + EPA and a Mediterranean diet). Outcomes: May improve neurocognitive outcomes in those with PCS, i.e., attention, memory, psychomotor speed, reaction time, executive function, etc.
Kemp et al. [36]	Participants: Community-dwelling adults with post-TBI sleep disturbance. Intervention: Melatonin 5 mg daily for 10 weeks versus amitriptyline. Outcomes: Melatonin may improve daytime alertness and alleviate symptoms of anxiety and depression.
Grima et al. [37]	Participants: Community-dwelling adults with post-TBI sleep disturbance. Intervention: Melatonin 2 mg daily for 10 weeks. Outcomes: Melatonin may improve sleep quality and symptoms of anxiety.
Theadom et al. [38]	Participants: Community-dwelling adults with persistent cognitive difficulties and history of mTBI. Intervention: Enzogenol^®^ 1000 mg daily for 16 weeks. Outcomes: Enzogenol^®^ may reduce cognitive shortcomings associated with chronic mTBI.
Walter et al. [39]	Participants: College-aged athletes with a history of SRCs and residual symptoms. Intervention: Enzogenol^®^ 1000 mg daily for 6 weeks. Outcomes: Enzogenol^®^ may reduce mental fatigue and improve self-reported sleep problems in college-aged athletes with a history of SRCs.
Rippee et al. [40]	Participants: Community-dwelling adults with PCS symptoms. Intervention: KD as 1:1 ratio of g lipid to g of non-lipid, and energy as 70–75% from fat. MCT oil ~1–2 tbsps. Outcomes: The KD may help to improve PCS symptoms; however, more clinically robust studies are required to support the findings from its feasibility study.
Theadom et al. [41]	Participants: Adults with chronic TBI experiencing cognitive difficulties. Intervention: MLC901 capsules 0.8 g, three times daily for 6 months. Outcomes: MLC901 may contribute to improved complex attention and executive functioning in those post-TBI.
Singh et al. [42]	Participants: Post-menopausal females with medically diagnosed PCS. Intervention: Self-administered varying dosing of CBD or a 20:1 CBD:THC formula. Outcomes: Improvement in systolic blood pressure volume.

Abbreviations (alphabetical order); CBD = cannabidiol, CRP = concussion recovery programme; DHA = docosahexaenoic acid; EPA = eicosapentaenoic acid; KD = ketogenic diet; MCT = medium chain triglyceride; mg = milligram; mTBI = mild traumatic brain injury; n-3FAs = omega-3 fatty acids; PCS = post-concussion syndrome; SRC = sports-related concussion; TBI = traumatic brain injury; THC = tetrahydrocannabinol. Outcome measurements.

**Table 6:** Black and Downs [43] for the 10 included studies. Filled circles indicate that the criteria were met, empty circles indicate the criteria were not met, diamonds indicate that the criteria were partially met and question marks indicate that was unable to be determined.

**Study**	**Q1**	**Q2**	**Q3**	**Q4**	**Q5**	**Q6**	**Q7**	**Q8**	**Q9**	**Q10**	**Q11**	**Q12**	**Q13**	
Amen et al. [34]	o	o	o	•	o	•	•	o	•	•	?	?	?	
Fotuhi et al. [35]	•	•	•	•	◊	•	•	o	o	•	o	o	•	
Kemp et al. [36]	•	•	•	•	o	•	•	•	•	•	o	?	•	
Grima et al. [37]	•	•	•	•	•	•	•	•	•	•	o	?	•	
Theadom et al. [38]	•	•	•	•	•	•	•	•	•	•	o	?	?	
Walter et al. [39]	•	•	•	•	o	•	•	o	o	o	?	?	•	
Rippee et al. [40]	•	•	•	•	o	•	•	•	•	•	o	?	•	
Theadom et al. [48]	•	•	•	•	•	•	•	•	•	•	•	?	•	
Singh et al. [42]	•	•	•	•	•	•	•	•	•	o	?	?	?	
**Study**	**Q14**	**Q15**	**Q16**	**Q17**	**Q18**	**Q19**	**Q20**	**Q21**	**Q22**	**Q23**	**Q24**	**Q25**	**Q26**	**Q27**
Amen et al. [34]	o	o	•	o	•	?	•	?	•	o	o	o	?	?
Fotuhi et al. [35]	o	o	•	•	•	?	•	?	?	o	o	?	?	?
Kemp et al. [36]	•	•	•	•	o	•	•	•	•	•	•	o	•	o
Grima et al. [37]	•	•	•	•	•	•	•	•	•	•	•	•	•	•
Theadom et al. [38]	•	•	•	•	•	•	•	•	•	•	•	•	•	?
Walter et al. [39]	•	•	•	•	•	o	•	•	?	•	•	o	?	?
Rippee et al. [40]	o	o	•	•	•	•	•	?	•	o	o	o	•	?
Theadom et al. [48]	•	•	•	•	•	•	•	•	?	•	•	•	•	o
Singh et al. [42]	o	o	•	•	•	?	•	•	•	o	o	?	•	o

## Appendix A

**Section and Topic**	**Item #**	**Checklist Item**	**Location Where Item Is Reported**
TITLE			
Title	1	Identify the report as a systematic review.	Cover page
ABSTRACT			
Abstract	2	See PRISMA 2020 for Abstract checklist.	P1
INTRODUCTION			
Rationale	3	Describe the rationale for the review in the context of existing knowledge.	P2–4
Objectives	4	Provide an explicit statement of the objective(s) or question(s) the review addresses.	P4
METHODS			
Eligibility criteria	5	Specify the inclusion and exclusion criteria for the review and how studies were grouped for syntheses.	P4–6
Information sources	6	Specify all databases, registers, websites, organisations, reference lists and other sources searched or consulted to identify studies. Specify the date when each source was last searched or consulted.	P4
Search strategy	7	Present the full search strategies for all databases, registers and websites, including any filters and limits used.	P4–6
Selection process	8	Specify the methods used to decide whether a study met the inclusion criteria of this review, including how many reviewers screened each record and each report retrieved, whether they worked independently and, if applicable, details of automation tools used in the process.	P6
Data collection process	9	Specify the methods used to collect data from the reports, including how many reviewers collected data from each report, whether they worked independently, any processes for obtaining or confirming data from study investigators and, if applicable, details of automation tools used in the process.	P6
Data items	10a	List and define all outcomes for which data were sought. Specify whether all results that were compatible with each outcome domain in each study were sought (e.g., for all measures, time points, analyses) and, if not, the methods used to decide which results to collect.	P4–6
	10b	List and define all other variables for which data were sought (e.g., participant and intervention characteristics, funding sources). Describe any assumptions made about any missing or unclear information.	P4–6
Study risk of bias assessment	11	Specify the methods used to assess the risk of bias in the included studies, including details of the tool(s) used, how many reviewers assessed each study and whether they worked independently and, if applicable, details of automation tools used in the process.	P7
Effect measures	12	For each outcome, specify the effect measure(s) (e.g., risk ratio, mean difference) used in the synthesis or presentation of results.	
Synthesis methods	13a	Describe the processes used to decide which studies were eligible for each synthesis (e.g., tabulating the study intervention characteristics and comparing against the planned groups for each synthesis (item #5)).	P4–6
	13b	Describe any methods required to prepare the data for presentation or synthesis, such as handling of missing summary statistics or data conversions.	
	13c	Describe any methods used to tabulate or visually display results of individual studies and syntheses.	
	13d	Describe any methods used to synthesize the results and provide a rationale for the choice(s). If meta-analysis was performed, describe the model(s), method(s) to identify the presence and extent of statistical heterogeneity and software package(s) used.	
	13e	Describe any methods used to explore possible causes of heterogeneity among the study results (e.g., subgroup analysis, meta-regression).	
	13f	Describe any sensitivity analyses conducted to assess robustness of the synthesized results.	
Reporting bias assessment	14	Describe any methods used to assess the risk of bias due to missing results in a synthesis (arising from reporting biases).	P7–9
Certainty assessment	15	Describe any methods used to assess certainty (or confidence) in the body of evidence for an outcome.	
RESULTS			
Study selection	16a	Describe the results of the search and selection process, from the number of records identified in the search to the number of studies included in the review, ideally using a flow diagram.	P8
	16b	Cite studies that might appear to meet the inclusion criteria but were excluded, and explain why they were excluded.	P8
Study characteristics	17	Cite each included study and present its characteristics.	P15–22
Risk of bias in studies	18	Present assessments of risk of bias for each included study.	
Results of individual studies	19	For all outcomes, for each study, present: (a) summary statistics for each group (where appropriate) and (b) an effect estimate and its precision (e.g., confidence/credible interval), ideally using structured tables or plots.	
Results of syntheses	20a	For each synthesis, briefly summarise the characteristics and risk of bias among contributing studies.	
	20b	Present results of all statistical syntheses conducted. If meta-analysis was performed, present each the summary estimate and its precision (e.g., confidence/credible interval) and measures of statistical heterogeneity. If comparing groups, describe the direction of the effect.	
	20c	Present results for all investigations of possible causes of heterogeneity among study results.	
	20d	Present results for all sensitivity analyses conducted to assess the robustness of the synthesized results.	
Reporting biases	21	Present assessments of risk of bias due to missing results (arising from reporting biases) for each synthesis assessed.	
Certainty of evidence	22	Present assessments of certainty (or confidence) in the body of evidence for each outcome assessed.	
DISCUSSION			
Discussion	23a	Provide a general interpretation of the results in the context of other evidence.	P32–34
	23b	Discuss any limitations of the evidence included in this review.	P34
	23c	Discuss any limitations of the review processes used.	P34
	23d	Discuss implications of the results for practice, policy and future research.	P34
OTHER INFORMATION			
Registration and protocol	24a	Provide registration information for this review, including register name and registration number or state that this review was not registered.	P4
	24b	Indicate where the review protocol can be accessed or state that a protocol was not prepared.	
	24c	Describe and explain any amendments to information provided at registration or in the protocol.	MA
Support	25	Describe sources of financial or non-financial support for this review, and the role of the funders or sponsors in this review.	P35
Competing interests	26	Declare any competing interests of the review authors.	P35
Availability of data, code and other materials	27	Report which of the following are publicly available and where they can be found: template data collection forms; data extracted from included studies; data used for all analyses; analytic code; and any other materials used in the review.	Available upon request
